# Ecotoxicity of Polyvinylidene Difluoride (PVDF) and Polylactic Acid (PLA) Microplastics in Marine Zooplankton

**DOI:** 10.3390/toxics10080479

**Published:** 2022-08-17

**Authors:** Michela Di Giannantonio, Chiara Gambardella, Roberta Miroglio, Elisa Costa, Francesca Sbrana, Marco Smerieri, Giovanni Carraro, Roberto Utzeri, Marco Faimali, Francesca Garaventa

**Affiliations:** 1 Early PostDoc Mobility Grant—Swiss National Science Foundation, 3000 Bern, Switzerland; 2 Institute for the Study of the Anthropic Impact and Sustainability in the Marine Environment (CNR-IAS), National Research Council, Via de Marini 16, 16149 Genova, Italy; 3 Institute of Biophysics (CNR-IBF), National Research Council, Via de Marini 16, 16149 Genova, Italy; 4 Schaefer SEE srl, Via Luigi Einaudi 23, 45100 Rovigo, Italy; 5 Institute of Materials for Electronics and Magnetism (CNR-IMEM), National Research Council, Via Dodecaneso 33, 16149 Genova, Italy; 6 Institute of Molecular Science and Technologies (CNR-SCITEC), National Research Council, Via de Marini 16, 16149 Genova, Italy

**Keywords:** behavior, cnidarians, crustacean, ecotoxicology, emerging contaminants, marine biota, novel detection method

## Abstract

The aim of this study was to investigate the ecotoxicity of polyvinylidene difluoride (PVDF) and polylactic acid (PLA) microplastics (MPs) in two marine zooplankton: the crustacean *Artemia franciscana* and the cnidarian *Aurelia* sp. (common jellyfish). To achieve this goal, (i) MP uptake, (ii) immobility, and (iii) behavior (swimming speed, pulsation mode) of crustacean larval stages and jellyfish ephyrae exposed to MPs concentrations (1, 10, 100 mg/L) were assessed for 24 h. Using traditional and novel techniques, i.e., epifluorescence microscopy and 3D holotomography (HT), PVDF and PLA MPs were found in the digestive systems of the crustaceans and in the gelatinous tissue of jellyfish. Immobility was not affected in either organism, while a significant behavioral alteration in terms of pulsation mode was found in jellyfish after exposure to both PVDF and PLA MPs. Moreover, PLA MPs exposure in jellyfish induced a toxic effect (EC50: 77.43 mg/L) on the behavioral response. This study provides new insights into PLA and PVDF toxicity with the potential for a large impact on the marine ecosystem, since jellyfish play a key role in the marine food chain. However, further investigations incorporating additional species belonging to other trophic levels are paramount to better understand and clarify the impact of such polymers at micro scale in the marine environment. These findings suggest that although PVDF and PLA have been recently proposed as innovative and, in the case of PLA, biodegradable polymers, their effects on marine biota should not be underestimated.

## 1. Introduction

Plastics are considered wonder products of the last century, and while their use has been essential to global development, they now represent a serious exotoxicity challenge. They pervade aquatic habitats because of their constantly increasing use, low cost, low weight, and high durability [1]. The annual global production of plastic has increased from 1.5 million tons in the 1950s to 360 million tons in 2018, and is projected to reach 2000 million tons by 2050 [2]. In addition, the ongoing COVID-19 pandemic has further increased the consumption of single-use plastics for medical purposes, including vast quantities of disposable face masks and viral tests, creating a new challenge for the scientific community [3]. The result is an ever-increasing amount of plastics and their production waste flowing from cities, ports, and industrial centers into the oceans [4,5,6].

Once in the environment, plastics of all sizes can be degraded into microplastics (MPs, 5 mm–0.001 mm) by biological, chemical, and physical factors [7]. In the aquatic environment, the action of UV light, salinity, wave action, and temperature change may promote plastic degradation into MPs [8]. One of the main consequences of their near-ubiquitous distribution in the marine environment is the unknown risk they pose to environmental health [9]. MPs have been reported in species throughout the aquatic food chain (i.e., crustaceans, mollusks, cnidarians, echinoderms, fish, and mammals), where they induce sub-lethal responses on fertilization, hatching, predatory ability, and swimming behavior [10,11,12,13,14,15]. Zooplankton species of marine vertebrates and invertebrates are no exception, and represent the base of the marine food chain. Many feed on phytoplankton and pass their energy upwards through the food web [16]. They mainly feed in surface waters, where MPs abundance is high, thus increasing the chances of encounter and ingestion [17]. Among zooplankton, MPs have been found ingested by crustaceans (e.g., copepods, barnacles, brine shrimps) [10,18,19,20] and jellyfish [21,22]. This ingestion is responsible for a reduction in fecundity, algal prey consumption, egg size and hatching success [23], and behavioral and enzyme activity alterations in crustaceans [24,25]. Conversely, MPs ingestion in jellyfish has only recently been investigated and proved responsible for altering physiology and overall fitness [21,26,27,28]. Jellyfish play a fundamental role in the ocean ecosystem, particularly in the trophic organization of the marine food webs. In fact, they directly prey on planktonic organisms, such as fish larvae and eggs, and compete with larvae and juvenile fish by feeding on their crustacean food source [29]. In turn, they are one of the main food sources for sea turtles, fish, and sea birds [30]. Moreover, jellyfish are becoming increasingly present in human diets to address overexploited traditional fisheries and could represent a direct vector for human ingestion of pollutants [31].

To begin to understand the full scope of this potential health hazard, this study investigates the uptake and the possible ecotoxicological effects of polyvinylidene difluoride (PVDF) and polylactic acid (PLA) MPs in two zooplankton species, the crustacean *Artemia franciscana*, also known as the brine shrimp (Kellogg, 1906), and the cnidarian *Aurelia* sp., or common jellyfish. These species represent two key organisms of the marine trophic chain and are prey and predator, respectively [32].

PVDF and PLA have been proposed as innovative and, in the case of PLA, biodegradable polymers [33,34]. In this regard, PVDF has been recently employed for face mask filtration due to its improved textile and biological properties [33]. PLA is considered an eco-friendly alternative to traditional plastics, is biocompatible with humans, and is employed in drug delivery and other biomedical applications [35].

The production of both materials has increased significantly in the last decades. The global production volume of PLA was around 190,000 tons in 2019 [36], while the global PVDF market was valued at USD 956 million in 2017, and it is expected to expand from 2018 to 2025 at an annual growth rate of 7.2% [37]. Both polymers can be used for several purposes, such as biomedical applications in drug delivery systems [25,38] or for producing a large variety of kitchen appliances, such as disposable tableware and cutleries [39].

PLA has attracted increasing attention because it is considered an eco-friendly alternative to traditional plastics, and is commonly obtained from renewable resources with low production costs [40]. For instance, it has become the most widely used filament polymer for 3D printing technologies [41], and has begun to replace environmentally detrimental polymers in monofilament fishing lines and netting for vegetation prevention [40]. Its degradation into innocuous lactic acid (within six months to two years) makes it ideal for medical implants that gradually transfer the load to the bones as the interested body area heals [42]. PLA is defined as the “polymer of the 21st century” by Balla et al. [43], and due to its superior mechanical and multifunctional properties that allow its use in various applications (i.e., biomedicine, additive technologies, packaging, fibers, automotive to agriculture [44,45,46,47]), it is a serious candidate to replace fossil-fuel-derived polymers. On the other hand, PVDF is a semicrystalline polymer that, thanks to its chemical resistance, mechanical strength, flexibility, and thermal stability, has been extensively employed in membrane technologies, such as water treatment [48]. Due to its smooth morphology and uniform pore structure, it also has found recent applications in manufacturing of nanofibrous filters for COVID-19 face masks [49].

**Figure 1:**
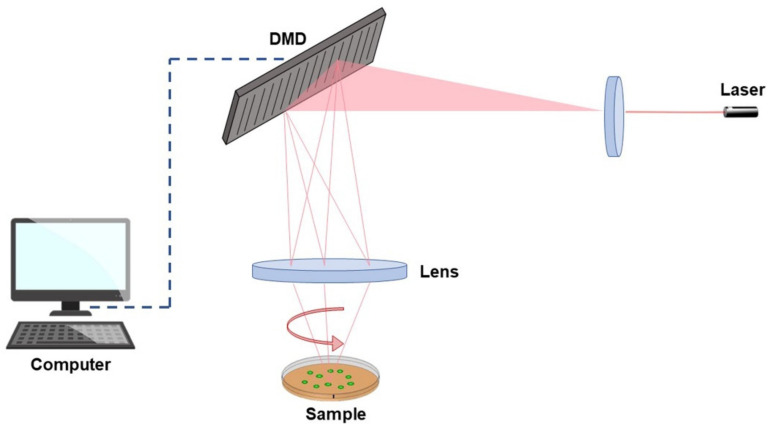
Representation of a Tomocube’s holotomography functioning.

Both PLA and PVDF can easily enter the aquatic environment and degrade into MPs, resulting in their recent study to monitor effects on marine biota. Studies have been conducted on microalgae [50], mollusks, and fish [51,52]; however, data on marine zooplankton are still scarce [53]. To fill this gap, the present study investigates the uptake and ecotoxicological effects of PLA and PVDF in zooplankton, particularly on brine shrimp and jellyfish. Internalization of MPs was investigated in brine shrimp nauplii and jellyfish ephyrae by both traditional and novel techniques, namely, epifluorescence microscopy and Tomocube’s holotomography (HT). The latter is an emerging system, recently applied in biology, consisting of a laser interferometric technique providing the 3D distribution of refractive indices (RI) characteristic of fixed and live cells as well as tissues [54]. It is a promising tool to obtain label-free 4D imaging, able to show details about the mechanisms and dynamics of cells, tissues, and subcellular organelles. Unlike conventional methods, such as phase contrast and differential interference contrast microscopy, HT does not need preparation steps, such as fixation, transfection, and staining, for environmental monitoring and in situ diagnostics [54,55]. As shown in Figure 1, the laser beam is split into two paths, the sample beam and the reference, that, when combined, generate a 2D hologram, recorded by a digital image sensor. The light through the sample will scatter differently based on the characteristic refractive indices of the various components. Exploiting a digital micro-mirror device, the light beam is rotated at various angles around the sample, and multiple 2D holograms are captured and then reconstructed into a 3D RI tomogram [56].

Despite its speed and ease of use, HT is still not common in ecotoxicology and environmental science. A preliminary work by Costa et al. [27] is one of the few examples to have used this technique to verify MP uptake in jellyfish ephyrae.

The scope of this study was to assess the ecotoxicity of PVDF and PLA MPs in *A. franciscana* and *Aurelia* sp. To achieve this goal, (i) MP uptake, (ii) immobility, and (iii) behavioral (swimming speed, pulsation mode) responses of brine shrimp larval stages and jellyfish ephyrae were assessed after exposure to PVDF and PLA MPs. The most sensitive stages of brine shrimp and jellyfish life-cycle towards traditional and emerging compounds [27,30,57,58,59,60], namely, instar I stage (nauplii) of *A. franciscana* and ephyra stage of *Aurelia* sp., were used for this study. Regarding behavioral responses, brine shrimp swimming speed and jellyfish frequency of pulsations, known to be very sensitive endpoints to assess MPs toxicity in zooplankton, were evaluated.

## 2. Materials and Methods

### 2.1. Plastic Materials and Grinding Process

Solvents and reagents used in this study were obtained at analytical grade; hence they were utilized without additional purification.

PVDF 1015 Solef^®^ (CAS: 24937-79-9) was supplied by Solvay Specialty Polymers (Bollate, Milan, Italy), with an average molecular weight of 573 kDa and polydispersity index of 2.4. The polymer was desiccated under vacuum at 60 °C for 6 h before use.

**Figure 2:**
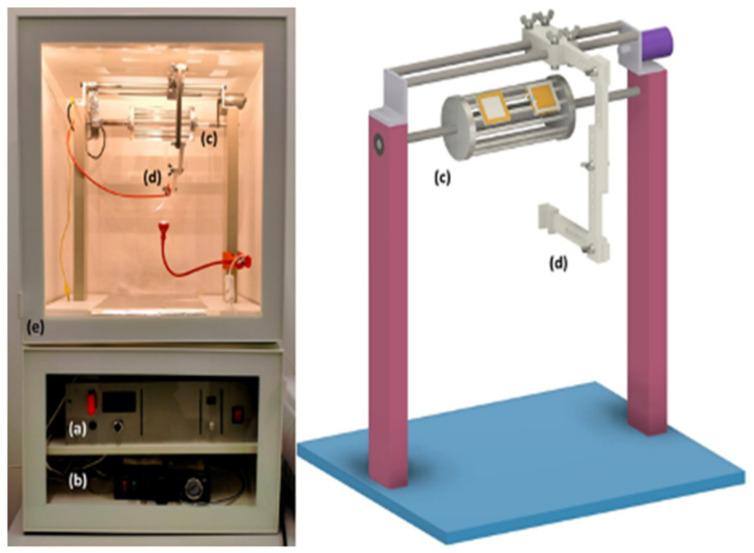
Electrospinning apparatus, consisting of (**a**) high-voltage power supply, up to 50 kV, (**b**) programmable syringe pump for syringes up to 50 mL, (**c**) aluminum cage collector (80 mm × 170 mm) that can spin up to 1k rpm, (**d**) needle positioning system, (**e**) MDF cabinet.

To fabricate PVDF nanofibers, a small batch of non-woven fabric was produced by electrospinning, following the protocol outlined in Russo et al. [61]. Electrospinning is a technique for producing nanofibers using the electrostatic force between a polymer solution droplet and a collector [62]. A solution was obtained by dissolving the PVDF powder in the mixture of solvents (dimethyl sulfoxide (DMSO, CAS: 67-68-5)/acetone 6:4, provided by Sigma Aldrich (St. Louis, MO, USA)) at 60 °C with an 8 wt% concentration. The solution was mixed for 30 min at 60 °C to facilitate the dissolution of the polymer. After a homogeneous solution was obtained it was degassed and left to cool to room temperature (25 °C, approximately 2 h). The solution was then electrospun using a self-built electrospinning setup, shown in Figure 2.

An electric field of about 1.2 kV/cm was applied between the syringe needle (spinneret) and the collector, kept at ground potential. The processing parameters used were a flow rate of 0.5 mL/h, a spinneret-to-collector distance of 10 cm, and an applied voltage of 12 kV. A collector rotation speed of about 500 rpm was used during the deposition. The apparatus was placed inside a dedicated cabinet to maintain a uniform deposition environment. The as-produced PVDF nanofiber mats were collected and dried on a heated plate at 60 °C for one hour in air. They were frozen in liquid nitrogen and ground with pestle and mortar inside a fume hood in order to obtain MPs (<500 μm in size). The powders were then stored in a vial.

PLA for 3D printers was purchased in a local shop, cut by scissors into small pieces (approximately 1 cm^2^), frozen in liquid nitrogen, and ground in a rotatory mill (Retsch ZM 1, Haan, Germany) to obtain MPs with a size of <500 μm.

#### 2.1.1. Morphological Characterization

After grinding, PVDF and PLA particle size and shape were characterized using a scanning electron microscope (SEM, Hitachi TM 3000, Tokyo, Japan), while the polymeric nature was checked by using a PerkinElmer Spectrum Two Fourier Transform Infrared Spectroscopy (FT-IR) spectrometer, equipped with Universal ATR (UATR) accessory with a 9- bounce diamond top-plate (Wave number range: 4000 and 450 cm^−1^; 4 cm^−1^ resolution; 32 scans). After measurements, the spectra were compared to reference spectra through libraries supplied by Perkin Elmer, with a >70% similarity threshold.

#### 2.1.2. Material Staining with Nile Red

Ground particles were labeled with Nile red according to Karakolis et al. with minor modifications [63]. Dry plastic particles were added to 100 μg/mL Nile red in a deionized water solution at a concentration of 50 mg of plastic particles per 10 mL of solution. To prepare this solution, 1 mg of Nile red was dissolved in 1 mL of acetone, and then the solution was added to 10 mL of deionized water. In order to maintain MPs in suspension, glass vials were left in an orbital shaker at 2 rpm speed for 24 h in darkness at room temperature. Plastics were then removed from the vial poured through a filter, and rinsed three times, each time being resuspended in fresh deionized water, poured through a filter, and then resuspended in filtered sea water (FSW). The following concentrations were used to detect PVDF and PLA uptake and to evaluate toxicity effects: 1, 10, and 100 mg/L.

### 2.2. Organisms

#### 2.2.1. *Artemia franciscana*

Certified dehydrated cysts of *A. franciscana* were purchased (MicroBioTests Inc., Belgium) and used for the experiments. Instar I stage nauplii were obtained by incubating 500 mg of cysts for 48 h at 28 °C under light source (3000–4000 lx) and continuous aeration of the cyst suspension in filtered seawater (FSW, 37‰ salinity). The hatched nauplii were separated from non-hatched cysts and then transferred with a Pasteur pipette into a beaker containing 0.22 µm FSW in a final concentration of 15–20 nauplii/mL.

#### 2.2.2. *Aurelia* sp.

Colonies of *Aurelia* sp. polyps attached on PVC tubes were obtained from the laboratories of the Aquarium of Genoa and transported to the laboratories of the Institute for the Study of the Anthropic Impact and Sustainability of the Marine Environment of the Italian National Research Council (CNR-IAS). They were placed in a thermostatic room at 20 °C in 1.5 L dark plastic tanks, filled with FSW (37‰ salinity), and gently aerated. Polyps were fed daily with *Artemia* sp. nauplii; seawater was changed every two days. Strobilation was induced by thermic shock and food starvation: PVC tubes with polyps were moved to 10 °C into 1.5 L dark plastic tanks filled with FSW; the polyps were not fed and seawater was not changed for one month. Once released by strobilation, ephyrae (0 days old) were immediately collected and used for the toxicity tests.

### 2.3. PVDF and PLA Uptake

Organisms were exposed to concentrations (0, 1, 10, 100 mg/L) of PVDF and PLA MPs individually for 24 h. Specifically, 10–15 *A. franciscana* nauplii were placed in each well of a 24-multi-well plate containing 1 mL of different material concentrations using an 80 µm mesh filter [24], while 8 *Aurelia* sp. ephyrae were individually placed directly in each well (2 mL of PLA and PVDF concentrations) to avoid interactions among organisms [30]. Organisms not exposed to MPs (0 mg/L concentration) were considered controls. For each concentration of PVDF and PLA, 4 replicates were prepared for ephyrae (8 ephyrae per replica, 24 ephyrae for each concentration). Afterward, they were incubated in the dark for 24 h at 25 °C and 20 °C for *A. franciscana* and *Aurelia* sp. ephyrae, respectively, according to previous studies [24,27]. For each concentration of PVDF and PLA, the organisms were washed three times with fresh FSW to remove any particles bound to the exoskeleton, according to Nasser and Lynch [64]. The organisms were anesthetized with menthol crystals and fixed in 4% paraformaldehyde solution in FSW. Brine shrimp nauplii and jellyfish ephyrae were then observed by an epifluorescence microscope (Olympus) and a 3D HT microscope (Tomocube Inc. model HT-2, Daejeon, South Korea), including the fluorescence module, according to Costa et al. [27]. A 3D map represents the 3D RI distribution of a sample and shows the different structures based on their different RI range value associated with the defined color map. No statistical analysis was performed on MP uptake.

### 2.4. Toxicity Tests

For ecotoxicological tests, brine shrimp nauplii and jellyfish ephyrae were exposed to concentrations (0, 1, 10, 100 mg/L) of PVDF and PLA individually for 24 h. Organisms were exposed to PVDF and PLA MPs in multi-well plates, and all tests were performed in quadruplicate to evaluate both immobility and behavioral responses after 24 h exposure. MP size range was not checked during the experiments. Regarding immobility, the analysis was performed under a stereomicroscope, and organisms that did not change their barycenter position and did not move their appendages in 5 s were referred to as “motionless”, according to Garaventa and colleagues [65]. Regarding behavioral responses, brine shrimps’ swimming speed and ephyrae frequency of pulsations were recorded by using an automatic recording system (Swimming Behavior Recorder—SBR), developed at CNR-IAS, set to record organisms’ movement for 3 s and 30 s in dark conditions for brine shrimps and ephyrae, respectively [24,30]. The experimental setup for measuring the behavioral responses is described in the works of Faimali et al. [30,66]. Data are referred to as average swimming speed mm/second or pulsation number/minute [30].

All data are expressed as means ± standard error of the 4 replicates. Effective concentration (EC50; MPs concentration resulting in 50% immobility, swimming speed, or pulsation effect in the exposed organisms after 24 h) and related 95% confidence limits were calculated using Trimmed Spearman Karber analysis [67]. Significant differences between controls and treated samples were determined using one-way analysis of variance (ANOVA) followed by a Tukey test. When data failed to meet the assumption of normality, the nonparametric Kruskal–Wallis test and Mann–Whitney test were used to compare individual treatments. Data were considered significantly different when *p* < 0.01. SPSS statistical software (Statistical Package for the Social Sciences, Version 20; New York, NY, USA) was used for data analysis.

## 3. Results

### 3.1. Material Characterization

**Figure 3:**
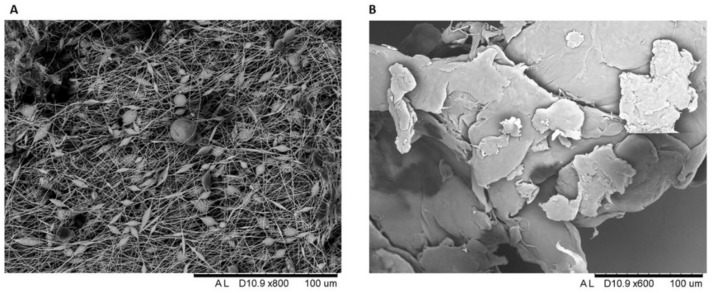
(**A**) SEM images of PVDF and (**B**) PLA after grinding.

Both polymers were characterized by SEM to analyze their morphology and their size (Figure 3A,B). As observed in Figure 3A, the electrospun PVDF fibers (50–500 µm in size) revealed a surface morphology with small beads placed along the nanofiber length, characterized by diameters ranging from 2.5 to 30 µm. On the other hand, PLA presented a fractured morphology with different range sizes (25–350 µm) with random cracks, as shown in Figure 3B.

The FTIR spectra proved a high percentage of purity for both materials: 94% for PVDF and 98% for PLA (Supplementary Figure S1A,B).

### 3.2. MPs Uptake

**Figure 4:**
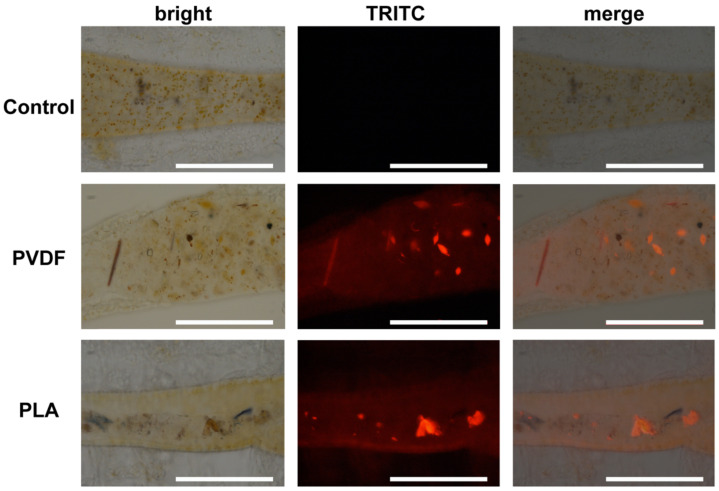
Epifluorescence of PVDF and PLA MPs stained with Nile red in the *A. franciscana* nauplii. Control refers to *A. franciscana* nauplii not exposed to MPs. After exposure to PVDF and PLA MPs, materials were localized in the gut. Bars equal 100 µm.

**Figure 5:**
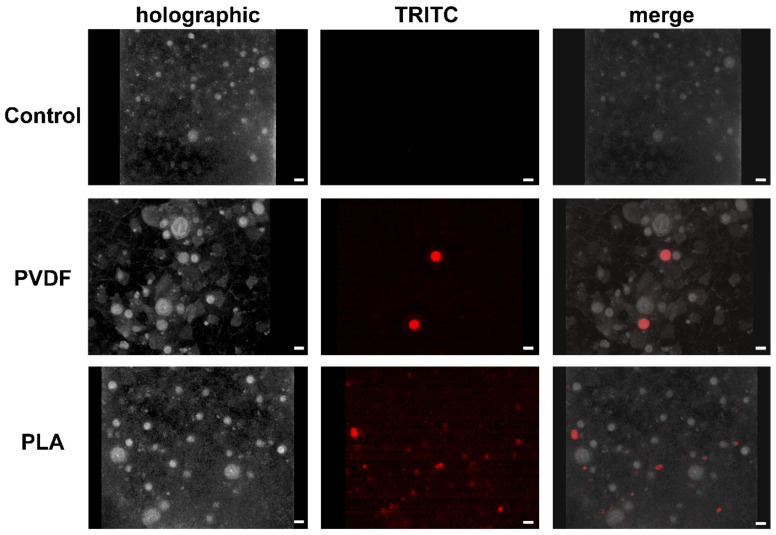
Epifluorescence of PVDF and PLA stained with Nile red in *Aurelia* sp. ephyrae jellyfish acquired together with holotomogram. Both materials (red color representing the fluorescence channel; refractive index 1.42 for PVDF and 1.4 for PLA) are localized inside the gelatinous body (index range 1.355–1.378) after 24 h exposure. Bars equal 30 μm.

The internalization of PVDF and PLA MPs was observed in the larval stage (nauplii) of the crustacean *A. franciscana* and in the cnidarian *Aurelia* sp. within 24 h of exposure (Figure 4 and Figure 5). For both polymers, the uptake occurred only at the highest concentration (100 mg/L). Fluorescently labeled PVDF and PLA MPs were localized in the crustacean gut and jellyfish gelatinous tissue by means of epifluorescence microscope and Tomocube’s HT.

Because of the thick gelatinous body of the ephyrae *Aurelia* sp., which prevents MPs detection through the epifluorescence microscope, HT was used (Figure 5). Moreover, a video of the 3D section of the analyzed organisms was produced (Supplementary Videos S1 and S2), where it is possible to localize MPs in the gelatinous body, among the nematocysts.

### 3.3. Ecotoxicology

**Figure 6:**
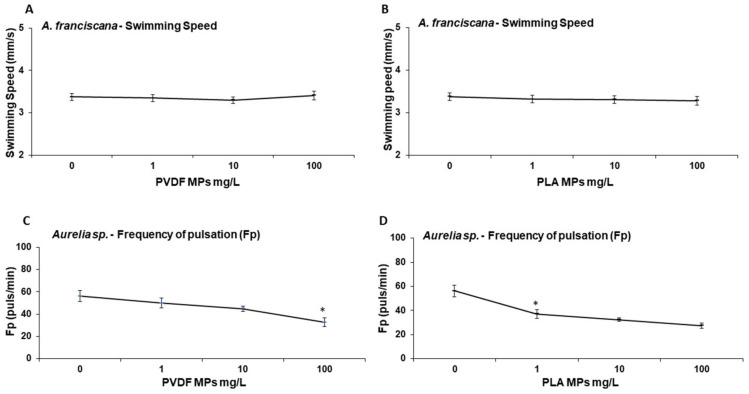
Behavioral responses of *A. franciscana* nauplii (**A**,**B**) and *Aurelia* sp. ephyrae (**C**,**D**) exposed to PVDF and PLA MPs (0, 1, 10, 100 mg/L) for 24 h. Swimming speed (mm/s) of *A. franciscana* after exposure to PVDF (**A**) and PLA (**B**) MPs. Frequency of pulsation of *Aurelia* sp. ephyrae after exposure to PVDF (**C**) and PLA (**D**). * *p* < 0.01.

Immobility was not affected in brine shrimp nauplii or in jellyfish ephyrae (<2%, data not shown). Behavioral responses of zooplanktonic species exposed to different concentrations (1, 10, 100 mg/L) of PVDF and PLA MPs are reported in Figure 6. After 24 h exposure to PVDF and PLA, brine shrimp mobility was not significantly affected, while jellyfish frequency of pulsation decreased significantly (*p*-value: 0.0158) at 100 mg/L and from 1 mg/L (*p*-value: 0.016) upwards for PVDF and PLA, respectively. Exposure to PLA permitted an EC50 calculation (EC50 = 77.43 (7.83–100) mg/L).

## 4. Discussion

In this work, PVDF and PLA MPs were obtained in order to investigate, for the first time, their potential uptake and the ecotoxicological effects in the larval stages of the crustacean *A. franciscana* and in the cnidarian *Aurelia* sp. Chemical characterization through FTIR confirmed the high purity of both polymers [68,69]. The fibers of PVDF obtained by electrospinning are characterized by beads of different sizes placed along the nanofiber length [70], while PLA is a more rigid and fragile material, as suggested by the presence of a fractured morphology [71,72]. Such data available in literature are confirmed by the present study, where micro-sized beads in PVDF and cracks in PLA morphology were found.

The size of the ground MPs detected through SEM analysis reveals that both materials at the micro size range (25–350 µm diameter) could easily be ingested by the organisms selected in this study. In fact, the internalization of PVDF and PLA MPs was demonstrated in brine shrimp nauplii and jellyfish ephyrae by using traditional and novel techniques. While epifluorescence microscopy allowed detection of MPs, as already reported for other MP polymer types in other crustaceans [19,73,74], this traditional method failed to detect high concentrations of investigated MPs in jellyfish. Jellyfish ephyrae are formed by a gelatinous tissue, constituted by thin epithelial cellular layers with complex morphologies in proximity to thick regions of the highly hydrated extracellular matrix [75]. This structure of the tissues of gelatinous marine invertebrates makes it difficult to verify particles’ internalization in the deep layers with traditional techniques (e.g., epifluorescence and confocal microscopy) [27,76]. MP uptake could be verified through HT though, suggesting a positive correlation between the current state of the research on HT and revealing MP ingestion in gelatinous zooplankton. Traditional MPs (i.e., polyethylene, PE) were also detected in the gelatinous tissue of *Aurelia* sp. by Costa et al. [27]. Although to date HT is still underutilized on living organisms, in the present study we promote the use of the technique in gelatinous zooplankton, since it allowed detection of both PVDF and PLA MP uptake in jellyfish ephyrae. Our results suggest its potential to be used in other organisms known for producing a large amount of mucus under different stress conditions, which may have prevented traditional techniques detecting particle internalization (e.g., nanoplastics, MPs) [77,78].

The use of plastic dyeing protocols can also facilitate MP detection in certain laboratory-based studies [79,80]. While dyes such as Nile Red do not successfully stain all polymers, e.g., PE, polypropylene, polystyrene, and polyurethane were easily detected in zooplankton [63], while polyvinylchloride, polyamide, and polyester were not. In the present study, we found that Nile red ameliorated PLA and PVDF MP detection, achieving greater selectivity and increasing the intensity of fluorescence of these two polymers with respect to optical microscopy techniques [63].

Using the above-mentioned techniques, we demonstrated PVDF and PLA MPs in zooplankton, specifically in the brine shrimp digestive system and jellyfish gelatinous tissue. PVDF uptake has previously been demonstrated in the marine biota, localized in the digestive system of mussels, clams, and oysters [51,52] and in the gills and muscles of commercial fish species [81] in wild caught species or at laboratory exposures, independently of the real or artificial conditions. Conversely, PLA has been only proved in ascidians during laboratory exposure [82], but no data are available on its localization in marine biota. Therefore, this study is the first one localizing, through a novel detection method, PLA MPs in a marine species. Although these MPs were internalized, they did not appear to affect organisms’ survival in the limited context of this study. These findings are in agreement with the literature, where exposure to biodegradable and non-degradable polymer MPs does not induce mortality in marine biota, as seen in sea urchins [53,83]. However, sub-lethal responses at high concentrations due to short-term exposure to either conventional or biodegradable MPs (e.g., PLA) may yet prove a threat to the marine ecosystem in some marine species. As we reported here, there was a significant behavior decrease in jellyfish ephyrae from 1 mg/L upward. These findings are also confirmed in the literature, where PLA short-term exposure inhibited marine algal growth at 10 and 50 mg/L concentrations [50], promoted changes in blue mussel hemolymph immunological profiles [84], and altered respiration rate and induced stress in the European flat oyster *Ostrea edulis* and in the lugworm *Arenicola marina* [84,85].

Contrary to PLA, no studies have been published so far on the ecotoxicity of PVDF MPs towards marine organisms. This study contributes to filling the gap by providing new insights into PVDF toxicity at high concentrations. Currently, detailed PLA and PVDF concentrations in the aquatic environment are unknown. However, considering that both PVDF and PLA MPs can be ingested in marine biota, causing sub-lethal effects at high concentrations, as shown here, research to quantify these materials in the marine environment is needed for an environmental risk assessment. This is a major goal of ecotoxicity studies in general [83], to link real environmental concentration of target materials with ecotoxicological data, allowing appropriate pollutant threshold determination to protect the marine biota and the human food chain.

The zooplankton species used in this study, brine shrimp and jellyfish, represent prey and predator, respectively, and offer a window into the effects of MPs on the food chain in general. The two selected species show no survival effects post MP exposure, while a different sensitivity in terms of behavioral response was observed. Although these polymers at micro size do not affect immobility after short exposure, it would be worth investigating their long-term effects to verify their eco-compatibility over time, not only in single species but also along a simplified two-level trophic chain formed by brine shrimp and jellyfish ephyrae.

Regarding the behavioral response, no effects on swimming speed were detected in the brine shrimp, while jellyfish frequency of pulsation changes confirmed the sensitivity of the ephyrae towards environmental stressors seen in previous studies, including pesticides, nanoparticles, and conventional MPs [27,30,59].

Jellyfish pulsation mode is significantly affected by contaminants, including MPs, at concentrations lower than 2–4 orders of magnitude compared to other marine organisms [27]. The presence of PVDF and PLA MPs in jellyfish gelatinous tissue may be responsible for affecting and modulating pulse frequency. In the gelatinous tissue there are specific structures of the nervous system, namely, rhopalia, that trigger ephyrae contractions [86], resulting in an alteration of the pulsation frequency. Moreover, PVDF and PLA MPs are localized inside the gelatinous body of *Aurelia* sp. ephyrae among the nematocysts, used to capture prey and to protect jellyfish from predators. This suggests that jellyfish may have ingested MPs wrongly recognized as food [21], since no food was provided during the present study [87].

The alteration of the frequency of pulsations due to the high concentration of PVDF and PLA MPs represents a potential significant risk in jellyfish’s ability to capture food, avoid predators, and maintain orientation in the water column [30]. In the natural environment, potential changes in jellyfish swimming activity caused by different stressors (i.e., contaminants) may have life-history consequences for jellyfish, influencing the dynamic of their bloom and the composition of the marine food web, since jellyfish are key components [88,89].

The EC50 value found in jellyfish ephyrae after PLA exposure highlights the toxicity of PLA MPs compared to PVDF MPs. Such results could be ascribed to the origin of the materials used in this study and to their different properties. Regarding the origin, commercial PVDF was purchased as virgin polymer and fabricated in this study through electrospinning, while PLA for 3D printers was purchased in a local shop as a final product. The latter could contain chemical additives and pigments sorbed onto plastics that may be released, inducing toxicological effects [60,90,91]. This may explain the different results found in jellyfish ephyrae, although chemical analysis should be performed to confirm this hypothesis. In addition, the higher toxicity levels (in terms of EC50) of PLA, compared to PVDF, may reside in the different chemical properties of the polymers. Indeed, the PVDF is considered a high-performance polymer, characterized by a pronounced chemical inertness, which may decrease the level of interaction with the live organisms, and thus the toxicity relative to PLA [92]. Additionally, their different mechanical properties may influence the different toxicity towards the marine organisms.

The literature data report the major toxicity of PLA compared to other biopolymers [93]. Moreover, PLA has been proved to be more toxic than traditional polymers towards marine biota. Li et al. found a higher growth inhibition rate in microalgae *S. costatum* exposed to PLA than polystyrene and polyethylene terephthalate, indicating that PLA is more toxic than those traditional polymers [50]. However, in the same study, the authors demonstrated that PLA was less toxic to microalgae than other traditional polymers, such as PE. Our findings on jellyfish confirm the lower toxicity of PLA MPs compared to those reported in the literature for PE MPs, in terms of EC50 or LOEC (lowest observed effect concentration—the lowest concentration where an effect has been observed in ecotoxicology) values (Table 1). Even the results on the crustacean *A. franciscana* highlight the lower toxicity of PLA than PE. In this regard, immobility and behavior were not affected at any PLA concentrations, while a significant swimming speed alteration was found from 0.01 mg/L PE MPs [94]. These findings suggest that toxicity may vary according to the polymer types and that biodegradable polymers are not always less toxic than traditional ones.

## 5. Conclusions

Through the use of traditional and novel techniques, PVDF and PLA MPs uptake was reported in two zooplanktonic species, *A. franciscana* and *Aurelia* sp. Such internalization did not affect the survival of either organism in the limited context of this study; however, jellyfish behavior, namely, the pulsation frequency, was significantly altered after exposure to high concentrations of these materials, with a potential impact on the marine ecosystem, since jellyfish are key components of the food web. These findings suggest that although PVDF and PLA have been separately proposed as innovative, biodegradable, and eco-friendly polymers, their effects on marine biota should not be underestimated. More studies aimed at determining the real concentrations of PVDF and PLA in the marine environment should be performed to identify the threshold not to exceed in order not to pose any risks to marine life and the human food chain. Considering the increasing production of both polymers in the last decades, future ecotoxicity assessment studies performed by using a battery of species belonging to different trophic levels (e.g., bacteria, microalgae, fish) and using PVDF and PLA MPs collected in the real environment are paramount to better understand and clarify the impact of such polymers at micro scale in the marine environment and, therefore, on human health.

## Figures and Tables

**Table 1:** LOEC and EC50 (mg/L) values with confidence limits reported in the literature for marine zooplankton exposed to polylactic acids (PLA) and polyethylene (PE) microplastics (MPs).

Organisms	Species	Endpoint	Polymer	LOEC (mg/L)	EC50 (mg/L)	References
Cnidarians	*Aurelia* sp.	Immobility	PLA	>100	>100	This study
Cnidarians	*Aurelia* sp.	Frequency of pulsations	PLA	1	77.43 (7.83–100)	This study
Cnidarians	*Aurelia* sp.	Frequency of pulsations	PE	0.1	3.16 (1.73–5.79)	[27]
Cnidarians	*Aurelia* sp.	Frequency of pulsations	PE	0.01	<0.01	[27]
Crustaceans	*Artemia franciscana*	Immobility	PLA	>100	>100	This study
Crustaceans	*Artemia franciscana*	Swimming Speed	PE	>100	>100	This study
Crustaceans	*Artemia franciscana*	Immobility	PE	>10	>10	[94]
Crustaceans	*Artemia franciscana*	Swimming Speed	PE	0.01	>10	[94]
Microalgae	*Skeletonema costatum*	Growth inhibition	PLA	10	>50	[50]
Microalgae	*Skeletonema costatum*	Growth inhibition	PE	5	>50	[50]

## Data Availability

Not applicable.

## Supplementary Materials

The following supporting information can be downloaded at: https://www.mdpi.com/article/10.3390/toxics10080479/s1, Supplementary Figure S1: FTIR spectrum of PVDF (A) and PLA (B); Video S1: 3D section of jellyfish ephyrae with PVDF MPs (stained with Nile red—red fluorescence) inside the gelatinous tissue (stained in yellow), among the nematocysts (depicted in green); Video S2: 3D section of jellyfish ephyrae with PLA MPs (stained with Nile red—red fluorescence) inside the gelatinous tissue (stained in yellow), among the nematocysts (depicted in green).
