# Supplementary material for: Ecotoxicity of Polyvinylidene Difluoride (PVDF) and Polylactic Acid (PLA) Microplastics in Marine Zooplankton
Source: Toxics. 2022 Aug 17;10(8):479. doi: 10.3390/toxics10080479 (PMC9416274; doi:10.3390/toxics10080479)
Supplement: Supplementary file 1 [file toxics-10-00479-s001.zip › toxics-1829390-supplementary.pdf]

toxics

Supplementary Materials

Ecotoxicity of Polyvinylidene Difluoride (PVDF) and Polylactic Acid (PLA) Microplastics in Marine Zooplankton

Michela Di Giannantonio [1,*], Chiara Gambardella [2,*], Roberta Miroglio [2], Elisa Costa [2], Francesca Sbrana [3,4], Marco Smerieri [5], Giovanni Carraro [5], Roberto Utzeri [6], Marco Faimali [2] and Francesca Garaventa [1]

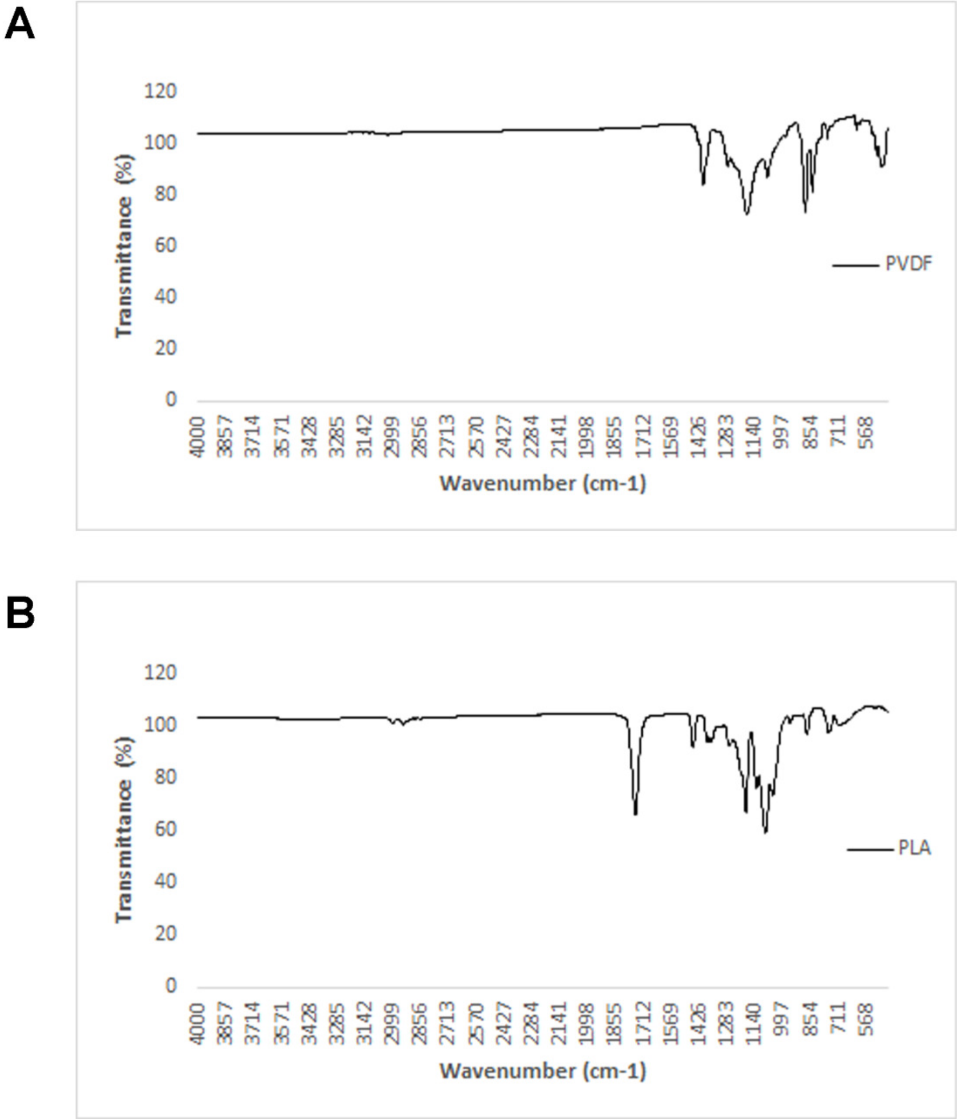

Figure S1. FTIR spectrum of PVDF (**A**) and PLA (**B**). Video S1: 3D section of jellyfish ephyrae with PVDF MPs (stained with Nile red—red fluorescence) inside the gelatinous tissue (stained in yellow), among the nematocysts (depicted in green). Video S2: 3D section of jellyfish ephyrae with PLA MPs (stained with Nile red—red fluorescence) inside the gelatinous tissue (stained in yellow), among the nematocysts (depicted in green).
